# Pyrite formation from FeS and H_2_S is mediated through microbial redox activity

**DOI:** 10.1073/pnas.1814412116

**Published:** 2019-03-18

**Authors:** Joana Thiel, James M. Byrne, Andreas Kappler, Bernhard Schink, Michael Pester

**Affiliations:** ^a^Department of Biology, University of Konstanz, 78464 Konstanz, Germany;; ^b^Geomicrobiology, Center for Applied Geoscience, Eberhardt Karls University of Tübingen, 72074 Tübingen, Germany;; ^c^Department of Microorganisms, Leibniz Institute DSMZ–German Culture Collection for Microorganisms and Cell Cultures, 38124 Braunschweig, Germany;; ^d^Institute for Microbiology, Technical University of Braunschweig, 38106 Braunschweig, Germany

**Keywords:** sulfur cycle, biogenic mineral transformation, syntrophy, deep biosphere, origin of life

## Abstract

The exergonic reaction of FeS with H_2_S to form FeS_2_ (pyrite) and H_2_ was postulated to have operated as an early form of energy metabolism on primordial Earth. Since the Archean, sedimentary pyrite formation has played a major role in the global iron and sulfur cycles, with direct impact on the redox chemistry of the atmosphere. However, the mechanism of sedimentary pyrite formation is still being debated. We present microbial enrichment cultures which grew with FeS, H_2_S, and CO_2_ as their sole substrates to produce FeS_2_ and CH_4_. Cultures grew over periods of 3 to 8 mo to cell densities of up to 2 to 9 × 10^6^ cells per mL^−1^. Transformation of FeS with H_2_S to FeS_2_ was followed by ^57^Fe Mössbauer spectroscopy and showed a clear biological temperature profile with maximum activity at 28 °C and decreasing activities toward 4 °C and 60 °C. CH_4_ was formed concomitantly with FeS_2_ and exhibited the same temperature dependence. Addition of either penicillin or 2-bromoethanesulfonate inhibited both FeS_2_ and CH_4_ production, indicating a coupling of overall pyrite formation to methanogenesis. This hypothesis was supported by a 16S rRNA gene-based phylogenetic analysis, which identified at least one archaeal and five bacterial species. The archaeon was closely related to the hydrogenotrophic methanogen *Methanospirillum stamsii*, while the bacteria were most closely related to sulfate-reducing Deltaproteobacteria, as well as uncultured Firmicutes and Actinobacteria. Our results show that pyrite formation can be mediated at ambient temperature through a microbially catalyzed redox process, which may serve as a model for a postulated primordial iron−sulfur world.

## Significance

Pyrite is the most abundant iron−sulfur mineral in sediments. Over geological times, its burial controlled oxygen levels in the atmosphere and sulfate concentrations in seawater. However, the mechanism of pyrite formation in sediments is still being debated. We show that lithotrophic microorganisms can mediate the transformation of FeS and H_2_S to FeS_2_ at ambient temperature if metabolically coupled to methane-producing archaea. Our results provide insights into a metabolic relationship that could sustain part of the deep biosphere and lend support to the iron−sulfur-world theory that postulated FeS transformation to FeS_2_ as a key energy-delivering reaction for life to emerge.

With an annual formation of at least 5 million tons, pyrite (FeS_2_) is the thermodynamically stable end product of iron compounds reacting with sulfide in reduced sediments, with the latter being produced mainly by microbial sulfate reduction. Consequently, pyrite is the most abundant iron−sulfur mineral on Earth’s surface (1). Over geological times, burial of pyrite was tightly intertwined with organic matter preservation in reduced sediments (2). These massive reservoirs of reduced sulfur and carbon are counterbalanced by the photosynthetically produced oxygen in Earth’s atmosphere (2). Despite this importance of pyrite for Earth’s iron, sulfur, and carbon cycles as well as Earth’s surface redox state, the mechanism of pyrite formation in natural environments is still being debated (1). Currently, two mechanisms are discussed to drive pyrite formation in sediments, which both preclude dissolution of precipitated iron(II) monosulfide (FeS) to an aqueous FeS intermediate. In the polysulfide pathway, FeS_aq_ is attacked by nucleophilic polysulfide to form FeS_2_ (Eq. **1**). Alternatively, pyrite may form from the reaction of FeS_aq_ with H_2_S (Eq. **2**), which is known as the H_2_S pathway or the Wächtershäuser reaction (1).

$$\mathrm{FeS}{+\text{S}}_{\text{n}}^{2-}\to {\text{FeS}}_{2}{+\text{S}}_{\text{n}-1}^{2-}\;\Delta {\text{G}}_{\text{r}}^{\;0\prime }=-64\text{to}-75\text{kJ}\cdot {\text{mol}}^{-1}$$ [1]

$${\text{FeS}+\text{H}}_{2}\text{S}\to {\text{FeS}}_{2}{+\text{H}}_{2}\;\;\Delta {\text{G}}_{\text{r}}^{0\prime }=-41k\text{J}\cdot {\text{mol}}^{-1}.$$ [2]

Using inorganic experimental systems, abiotic pyrite formation has been observed at temperatures of 25 °C to 125 °C (e.g., refs. 1 and 3). However, already, trace amounts of organic compounds containing aldehyde groups were reported to inhibit pyrite formation in such experiments (1, 4). Absence of stringent control for such compounds may explain why many other studies with abiotic systems did not observe pyrite formation at environmentally relevant temperatures (5–11). On the other hand, pyrite formation is known to take place in the presence of organic matter and especially of living microorganisms in sediments (5, 12). Indeed, pyrite formation could be observed in pure and enrichment cultures of sulfur-dismutating bacteria, with the assumption that these microorganisms provide mainly H_2_S as a substrate for concomitant abiotic pyrite formation (13–15). In addition, a more complex involvement of microorganisms has been postulated that includes the support of nucleation of FeS minerals on bacterial cell surfaces (16).

Pyrite formation according to Eq. **2** provides reducing equivalents in the form of H_2_ that could be coupled to the reduction of CO_2_ to CH_4_ or more complex organic matter. Coupling of pyrite formation to methanogenesis has been proposed by Jørgensen and coworkers (17) to be part of a cryptic sulfur cycle in deep marine sediments where it could support the enigmatic life forms of the deep biosphere. Coupling of this reaction to the synthesis of organic matter is the basis of the “iron−sulfur world” theory proposed by Wächtershäuser, by which pyrite formation is viewed as the central process that led to the transition from Fe–S surface-catalyzed synthesis of organic molecules to actual life on primordial Earth (18–20). Here, we show that the overall conversion of FeS with H_2_S to form FeS_2_ may supply microorganisms with energy for lithotrophic growth if syntrophically coupled to methanogenesis.

## Results and Discussion

### Pyrite Formation from FeS and H_2_S at 28 °C Relies on Active Microorganisms.

**Fig. 1.**
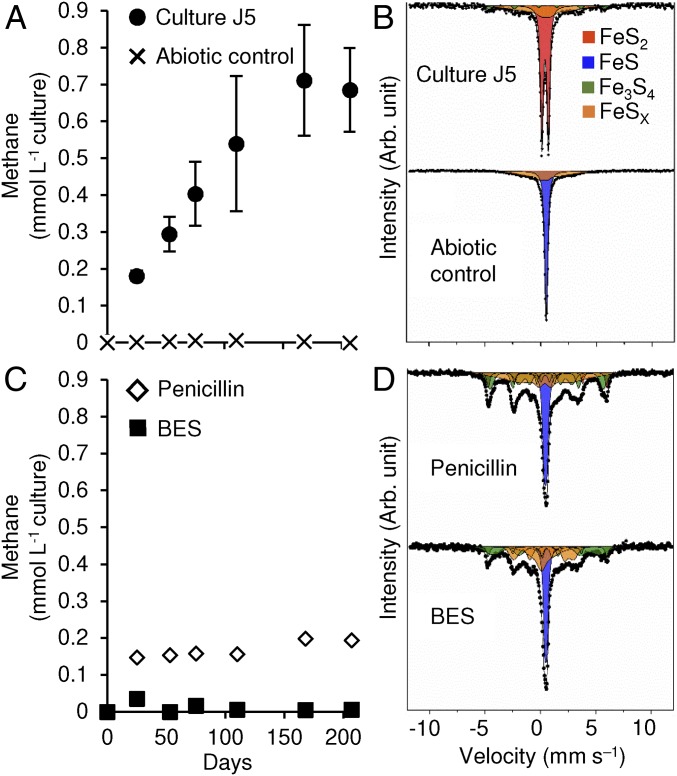
Time-resolved CH_4_ formation in comparison with iron−sulfur mineral composition after nearly 7 mo of incubation (207 d) in culture J5 compared with abiotic controls and incubations of culture J5 with penicillin-G (1,000 U⋅ml^−1^) or 2-bromoethanesulfonate (BES, 10 mM). (*A* and *C*) The mean ± SD of CH_4_ measurements of three independent incubations. SDs are often smaller than the actual symbol size. (*B* and *D*) Mössbauer spectra corresponding to the last time point in the presented time series, with FeS_2_ in red, FeS in blue, Fe_3_S_4_ in green, and intermediate FeS_x_ phases in orange. Black dots represent the measured raw data. Corresponding black lines represent the sum of all fitted mineral phases.

Mineral medium containing 5 mM FeS and 6 mM H_2_S as sole substrates and CO_2_/HCO_3_^−^ as carbon source was inoculated with digested sewage sludge, freshwater sediment, or marine sediment (*SI Appendix*, Table S1) and incubated at 16 °C or 28 °C. Microbial activity was followed via methane formation, and transfers were made every 3 mo to 8 mo, typically when the methane content in the headspace approached a plateau. Of seven enrichments started, four exhibited methane formation for more than 10 transfers. The most active enrichment culture, J5, which was started from digested sewage sludge and incubated at 28 °C, was characterized in more detail after more than 20 transfers. On average, the methane content reached 0.7 mmol per L of culture in J5. In contrast, no methane formation was observed in abiotic controls (Fig. 1*A*). This was mirrored in the turnover of total H_2_S: While in culture J5, total H_2_S decreased over time from approximately 6 mmol per L to a range of 0.04 mmol per L to 1.1 mmol per L of culture (*SI Appendix*, Fig. S1), noninoculated abiotic controls showed a much less pronounced decline of total H_2_S (3.7 mmol residual H_2_S per L). The observed decrease in the abiotic controls may be due to inorganic background reactions (see below).

Conversion of FeS solids was followed by ^57^Fe Mössbauer spectrometry. After nearly 7 mo of incubation, the Mössbauer spectrum of culture J5 was dominated by an FeS_2_ doublet (Fig. 1*B*), which corresponded to 53 to 63% of the iron−sulfur mineral phase (*SI Appendix*, Table S2). In contrast, no evidence of a singlet peak corresponding to FeS was present. In addition, a poorly defined sextet feature was required to achieve a satisfactory fit of the Mössbauer spectrum. This poorly defined sextet is best described as a metastable phase, which we have termed FeS_x_ in accordance with the notation used by Wan et al. (21), and represented 31 to 39% of the remaining iron−sulfur minerals. Furthermore, a well-defined sextet with a hyperfine magnetic field of 32 T was required to fit the data, which corresponded well to greigite (Fe_3_S_4_) (22) and made up 7 to 8% of the remaining iron−sulfur minerals. Greigite is the sulfur isomorph of magnetite and was previously observed as an intermediate phase in the FeS conversion to pyrite in abiotic studies (23, 24).

**Fig. 2.**
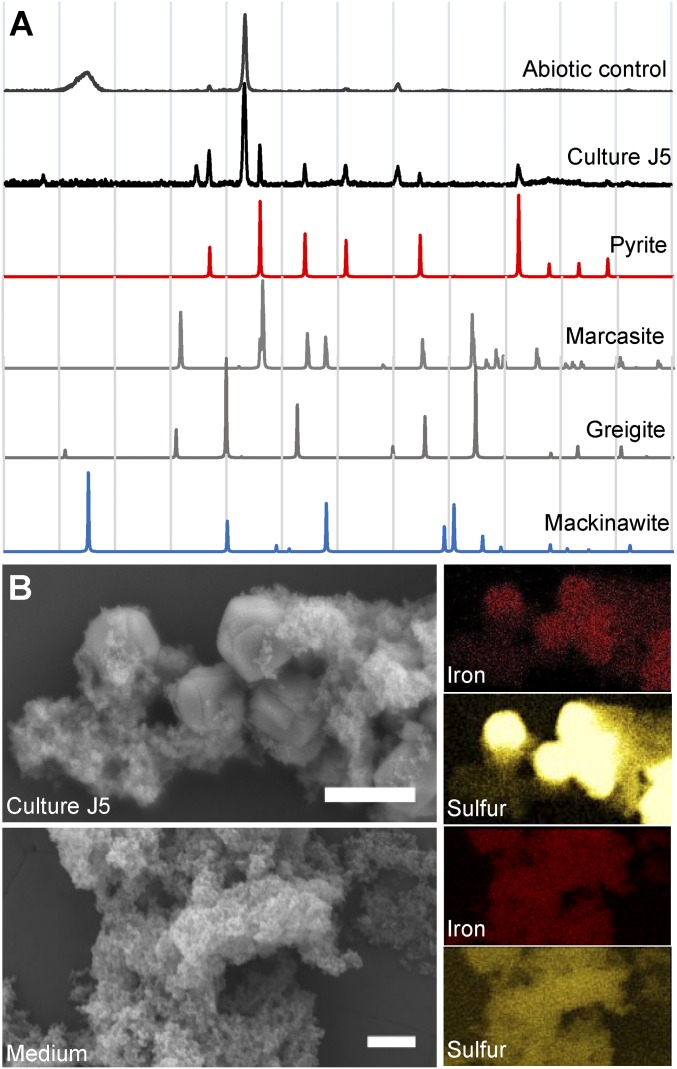
(*A*) Representative X-ray diffractograms of mineral precipitates formed in culture J5 and an abiotic control setup after 9 mo of incubation (281 d). Diffraction data of the two FeS_2_ dimorphs pyrite and marcasite as well as of Fe_3_S_4_ (greigite) and FeS (mackinawite) are given as reference. (*B*) (*Left*) Scanning electron microscopy images of a nearly 7-mo-old (211 d) culture J5 in comparison with freshly prepared medium without inoculum. (Scale bar, 2 µm.) (*Right*) The corresponding results from EDX (images correspond to a twofold down-scaling of the SEM images to the left). Besides atoms from medium salts, iron and sulfur were the only elements discovered in the mineral phases.

FeS_2_ formation in culture J5 was confirmed by X-ray diffraction (XRD) analysis, which recovered all major XRD reflections of pyrite in the obtained XRD pattern (Fig. 2*A*). Since no indication of a parallel formation of the dimorph marcasite was evident from the XRD pattern, the recovered FeS_2_ phase is referred to as pyrite from here on. Further support for pyrite formation in culture J5 was provided by scanning electron microscopy (SEM) coupled to energy-dispersive X-ray (EDX) spectroscopy (SEM-EDX). Here, micrometer-scale iron−sulfur crystals with a euhedral structure as typical of pyrite could be observed (Fig. 2*B*), which resembled the expected Fe:S ratio of 1:2 as revealed by EDX point measurements (*SI Appendix*, Fig. S2).

In contrast to culture J5, the Mössbauer spectrum of the nearly 7-mo-old noninoculated abiotic control was dominated by a prominent FeS singlet peak (64% of iron−sulfur minerals). In addition, a poorly defined sextet corresponding to FeS_x_ was required to achieve a satisfactory fit of the obtained data (36% of iron−sulfur minerals) (Fig. 1 and *SI Appendix*, Table S2). The abiotic conversion of FeS to FeS_x_ most likely explains the observed decrease of total H_2_S in the abiotic control. Absence of pyrite formation in abiotic controls was further supported by the obtained XRD patterns (Fig. 2*A*). In addition, freshly prepared medium was also devoid of pyrite as evidenced by an overall disordered iron−sulfur mineral phase in SEM-EDX images without any euhedral crystals indicative of pyrite (Fig. 2*B* and *SI Appendix*, Fig. S2).

**Fig. 3.**
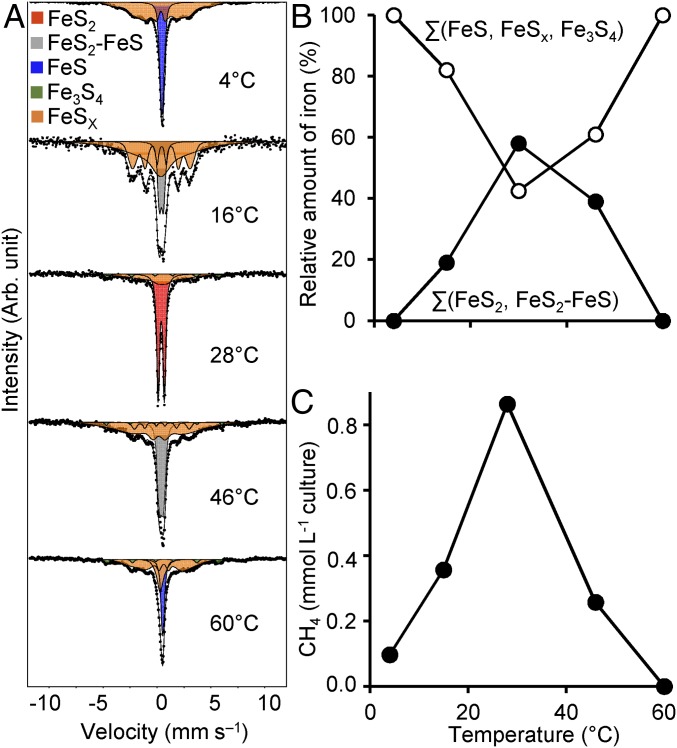
Temperature-dependent pyrite and methane formation in culture J5 after nearly 7 mo of incubation (207 d). (*A*) Mössbauer spectra showing the temperature-dependent iron−sulfur mineral composition (FeS_2_ in red, an intermediate FeS_2_−FeS phase in gray, FeS in blue, Fe_3_S_4_ in green, and intermediate FeS_x_ phases in orange). Black dots represent the measured raw data. Corresponding black lines represent the sum of all fitted mineral phases. (*B*) Relative abundance of pyrite in comparison with all other measured iron−sulfur minerals plotted against temperature as the explanatory variable. Details are provided in *SI Appendix*, Table S2. (*C*) Average amount of methane (*n* = 2) plotted against temperature as the explanatory variable.

To support our hypothesis of microbial involvement in pyrite formation in culture J5, we followed its iron−sulfur mineral transformation over a temperature gradient of 4 °C to 60 °C. The maximum of pyrite formation was evident at 28 °C. Incubation at lower (16 °C) or higher (46 °C) temperatures resulted in the formation of an as yet undefined “intermediate FeS_2_−FeS phase”, while no pyrite was formed at 4 °C and 60 °C (Fig. 3 *A* and *B* and *SI Appendix*, Table S2). The observed temperature profile of pyrite formation is typical of biologically catalyzed reactions centered on an optimum reaction temperature. In contrast, abiotic pyrite formation at temperatures of <100 °C was shown to follow a sigmoidal temperature dependence (3).

To rule out biologically supported abiotic pyrite formation, for example, by cell surfaces serving as passive catalysts, we used defined mixtures of either living or autoclaved microorganisms as inoculum. Despite using cell densities 10- to 100-fold higher in comparison with culture J5, neither methane nor pyrite formation was observed within a month’s period (*SI Appendix*, Fig. S3).

### Microbially Mediated Pyrite Formation Depends on Coupling to Methanogenesis.

Methane formation closely followed the temperature-dependent activity profile of pyrite formation (Fig. 3*C*), suggesting metabolic coupling of both processes. This hypothesis was further explored in an inhibition experiment using either penicillin G as a generic inhibitor of bacterial cell wall synthesis or 2-bromoethanesulfonate (BES) as a specific inhibitor of hydrogenotrophic methanogenesis (25). BES inhibited both methane and pyrite formation completely (Fig. 1 *C* and *D*). We interpret this as a direct coupling of microbially mediated pyrite formation to hydrogenotrophic methanogenesis. In support of this hypothesis, penicillin inhibited both pyrite and methane formation as well (Fig. 1 *C* and *D*). Here, methane formation ceased after an initial production of 0.15 mmol per L of culture. The latter is explained by penicillin’s mode of action, which inhibits growth of bacteria but not their initial metabolic activity. Further support for coupling of microbially mediated pyrite formation to methanogenesis was provided by a third inhibition experiment in which penicillin addition was supplemented by 79% H_2_ in the headspace. Also here, pyrite was not formed (*SI Appendix*, Table S2), while methane production was stimulated more than 10-fold by the added H_2_ (>10 mmol per L of culture). This clearly showed that methanogenesis could be uncoupled from pyrite formation and is essential for the latter. A likely explanation would be to keep H_2_ or another electron carrier at a low level to make microbially mediated pyrite formation energetically more favorable as typically observed in syntrophic processes (26). Coupling of pyrite formation to methanogenesis would result in a pyrite-to-methane ratio of 4:1 (Eq. **5**).

$$4\text{FeS}+4{\text{H}}_{2}\text{S}\to 4{\text{FeS}}_{2}+4{\text{H}}_{2}\;\;\left(\Delta {\text{G}}_{\text{r}}^{0\prime }=-164\text{kJ}/4\;{\text{mol FeS}}_{2}\right)$$ [3]

$${\text{CO}}_{2}+4{\text{H}}_{2}\to {\text{CH}}_{4}+2{\text{H}}_{2}\text{O}\;\;\left(\Delta {\text{G}}_{\text{r}}^{0\prime }=-131\text{kJ}/\text{mol}{\text{CH}}_{4}\right)$$ [4]

$$4\text{FeS}+4{\text{H}}_{2}{\text{S}+\text{CO}}_{2}\to 4{\text{FeS}}_{2}{+\text{CH}}_{4}+2\;{\text{H}}_{2}\text{O}\;\;\left(\Delta {\text{G}}_{\text{r}}^{0\prime }=-295\text{kJ}/{\text{mol CH}}_{4}\right).$$ [5]

Indeed, the ratio of pyrite to methane formed in culture J5 was 4.1:1 and 3.2:1 in two independent experiments at 28 °C (*SI Appendix*, Table S2), thus confirming the proposed overall reaction stoichiometry.

### Pyrite Formation Supports Microbial Growth.

**Fig. 4.**
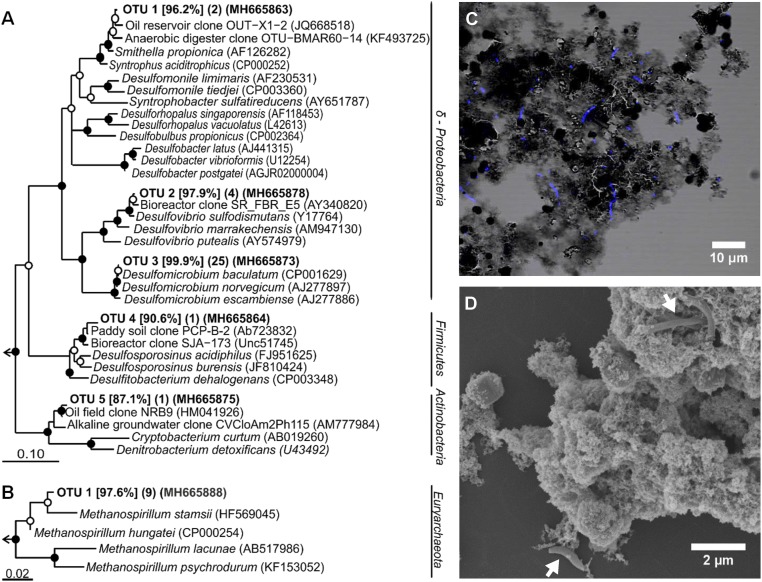
Bacterial and archaeal community composition of enrichment culture J5. RAxML trees based on (*A*) bacterial and (*B*) archaeal 16S rRNA gene sequences obtained from culture J5. Representative sequences of OTUs at the approximate species level (99% sequence identity) are shown. Sequence identity to the next cultured relative is given in percent in square brackets. Numbers of clones from the same OTU are presented in parenthesis followed by the GenBank accession number of a representative sequence. Bootstrap support is indicated by closed (≥90%) and open (≥70%) circles at the respective branching points. [Scale bar, 10% (Bacteria) and 2% (Archaea) estimated sequence divergence.] (*C*) Combined phase contrast image and fluorescent image of DAPI-stained cells and (*D*) scanning electron microscopy image with cells indicated by white arrows of culture J5 after 7.4 and 10 mo of incubation, respectively.

Culture J5 was transferred more than 20 times on medium containing FeS, H_2_S, and CO_2_ as sole substrates. This indicates a strict dependence on microbially mediated pyrite formation coupled to methanogenesis. Within incubation periods of 83 d to 248 d, cell densities increased by more than one order of magnitude, from 2 × 10^5^ cells per mL to 2 to 9 × 10^6^ cells per mL (*SI Appendix*, Fig. S4). Cells were typically found to be attached to mineral surfaces (Fig. 4*C*); however, there was no indication of cell encrustation (Fig. 4*D*). Assuming an average cell volume of about 1 μm^3^ and a dry mass content of 33% (27), our measured cell densities correspond to a maximum of *ca*. 3 mg dry cell mass per L. If formation of ATP requires 60 kJ⋅mol^−1^ to 70 kJ⋅mol^−1^ (28) and if–under ideal growth conditions–10.5 g biomass (dry weight) can be synthesized at the expense of 1 mol ATP (29), complete conversion of 5 mM FeS + 5 mM H_2_S according to Eq. **5** could yield 4 mM to 5 mM ATP or *ca*. 50 mg dry cell mass per L. Of course, the conditions of lithoautotrophic growth in our enrichment cultures were entirely different from those used in the growth experiment by Bauchop and Elsden (29), with heterotrophic growth in an organically rich medium. Moreover, formation of pyrite from FeS is an extremely slow process catalyzed at or close to mineral surfaces, which implies that major amounts of metabolic energy have to be invested into cell maintenance without concomitant growth (30). Thus, it is not surprising that our cell yields are lower than estimated above. If every partial reaction in Eq. **5** (five in total: four with FeS transformation and one with CH_4_ formation) uses about 20 kJ for synthesis of 1/3 of an ATP equivalent (28), the total process could synthesize 5/3 mmol ATP equivalents per liter. Considering that, in culture J5, a maximum of 62.5% FeS conversion to FeS_2_ was observed (*SI Appendix*, Table S2), the expected cell yield would–optimally–be 12 mg cell dry matter per L, which is close to the measured cell yield.

The community composition of culture J5 was analyzed by 16S rRNA gene clone libraries. All 16S rRNA gene sequences derived from amplification with a universal archaeal primer set belonged to the same species-level operational taxonomic unit (OTU, 99% sequence identity) and showed 97.6% sequence identity to *Methanospirillum stamsii* (Fig. 4*B*), a hydrogenotrophic methanogen isolated from a low-temperature bioreactor (31). Methanogenesis in culture J5 is likely performed by this OTU. Using a universal bacterial primer set, we detected five bacterial OTUs (Fig. 4*A*). The majority of clones (76%) belonged to OTU 3, which showed 99.9% sequence identity with *Desulfomicrobium baculatum*, a sulfate reducer within the Deltaproteobacteria (32, 33). Further OTUs related to Deltaproteobacteria were OTU 2 (12% relative abundance) and OTU 1 (6% relative abundance). OTU 2 was closely related to *Desulfovibrio sulfodismutans* (97.9% sequence identity), which is capable of dismutating thiosulfate or sulfite (34), while OTU 1 was distantly related (96.2% sequence identity) to *Smithella propionica,* which is known to degrade propionate in syntrophy with methanogens (35). The remaining two bacterial OTUs were either distantly related (<91% sequence identity) to cultured members of the Firmicutes (OTU 4, 3% relative abundance) or Actinobacteria (OTU 5, 3% relative abundance). Interestingly, all OTUs belonging to the Deltaproteobacteria and Firmicutes fell into larger clusters that include cultured representatives with a sulfur-related energy metabolism.

An exciting question remaining is whether pyrite formation is directly coupled to energy conservation in members of culture J5. These microorganisms could potentially utilize the exergonic H_2_S pathway (Eq. **2**), that is, the direct formation of FeS_2_ and H_2_ from FeS and H_2_S, for energy conservation. Alternatively, the actual energy metabolism could be restricted to sulfide conversion to zero-valent sulfur and H_2_ (Eq. **6**, where [S°] means any form of zerovalent sulfur, e.g., as part of a polysulfide chain or organically bound S° in the cell periphery). The latter would represent a reversal of the well-known sulfur respiration with H_2_ as, for example, *Wolinella succinogenes* catalyzes it (36).

$${\text{H}}_{2}\text{S}\to \left[{\text{S}}^{\text{o}}\right]+{\text{H}}_{2}\;\left(\Delta {\text{G}}_{\text{r}}^{\text{o}\prime }=+28\text{kJ}/\text{mol}\;\left[{\text{S}}^{\text{o}}\right]\right).$$ [6]

However, this reaction is endergonic under standard conditions. At the supplied concentration of H_2_S (5 mM), it would only become exergonic enough for energy conservation (−11 kJ/mol) (37, 38) if both the hydrogen partial pressure is kept below 10^−4^ bar as known for syntrophic cocultures involving methanogens (26) and zero-valent sulfur does not accumulate beyond 1 µM, for example, by effective removal by the polysulfide pathway (Eq. **1**) to finally produce pyrite. Thus, the reversal of sulfur oxidation would strictly depend on both a hydrogen-scavenging process, for example, methanogenesis, and a zero-valent sulfur-scavenging process, for example, the pyrite-forming polysulfide pathway. Whether the polysulfide pathway in this scenario would need active catalysis at the cell surface to allow for efficient removal of zero-valent sulfur or proceeds as a mere abiotic reaction remains open.

## Conclusion

Pyrite is produced in massive quantities in today’s sediments (2). However, its formation in nature is far from being understood, especially because its nucleation is kinetically hindered (1). The presence of sulfide-producing microorganisms as passive pyrite nucleation sites indicated support for abiotic pyrite formation (13–16), but could not be reproduced in every bacterial model system (6, 39). Our data are consistent with microorganisms being able to mediate the overall conversion of FeS and H_2_S to FeS_2_. Importantly, control experiments conclusively demonstrated that pyrite formation is not merely catalyzed by biomass components alone, such as the cell surface. A key next step will be the identification of the microorganisms that mediate pyrite formation in culture J5 and whether these microorganisms depend on pyrite formation as a novel form of energy metabolism or as a mere (abiotic) sink of zero-valent sulfur to drive sulfide oxidation as the energy-conserving step. Since we found only one archaeal species closely related to methanogens in our enrichments, it is likely that one or several of the bacterial partners are actually involved in FeS conversion to pyrite.

Our results further show that the reducing equivalents released from FeS transformation to pyrite can be transferred to methanogenesis. This opens an interesting perspective on the metabolic versatility sustaining the vast deep biosphere inhabiting Earth’s subsurface (40). While recalcitrant organic matter or H_2_ released by radiolytic cleavage of water (41, 42) have been proposed to sustain the enigmatic life forms of the deep biosphere, there is also experimental evidence of a cryptic sulfur cycle within deep sediments that would include pyrite formation coupled to methanogenesis to be functional (17). Our results show that this missing link could indeed be mediated by microorganisms and supply energy to support microbial growth. Since the redox potential (E_h_°′) of the FeS_2_/(H_2_S + FeS) couple is −620 mV at circumneutral pH (43), it is also well suited to provide reducing equivalents for CO_2_ fixation to acetate (E_h_°′ = −290 mV) and more complex organic matter. Wächtershäuser proposed in his “iron−sulfur world” theory that exactly this mechanism was the basis for an autocatalytic metabolism and the resulting evolution of life at hydrothermal vents on primordial Earth (e.g., refs. 20 and 44). Our enrichment cultures may serve as a model to elucidate the chemical and biochemical basis of this hypothesis.

## Methods

### Cultivation.

Enrichment cultures were initiated and maintained in carbonate-buffered, sulfide-reduced (1 mM) freshwater mineral medium (45) supplemented with selenite−tungstate solution (46), seven-vitamin solution (45), and trace element solution SL10 (47). The medium was prepared and stored under an N_2_/CO_2_ atmosphere (80:20). The final pH was adjusted to 7.2 to 7.4. From a CO_2_-neutralized sulfide stock solution (48), 350 µmol H_2_S was added to 70 mL of mineral medium in 180-mL serum bottles that were sealed with butyl rubber stoppers. Since 1 mM H_2_S was already present as reducing agent in the mineral medium, the final amount of H_2_S was 420 µmol. FeS was prepared from anoxic solutions of 0.4 M FeCl_2_ and 0.4 M Na_2_S. The resulting FeS precipitate was washed at least once and resuspended in oxygen-free distilled water. For Mössbauer spectroscopy analysis, FeS was prepared from an FeCl_2_ solution that contained 10% ^57^FeCl_2_ to enhance signal quality. Then 350 µmol of FeS was added to 70 mL of mineral medium. Enrichment cultures were incubated in the dark at 28 °C if not indicated otherwise. For inhibition experiments, cultures were supplemented with either penicillin-G (1,000 U⋅mL^−1^) or 2-bromoethanesulfonate (10 mM). Abiotic controls were run without inoculum.

### Monitoring of Substrate Turnover.

For total dissolved sulfide measurements [Σ(H_2_S_aq_, HS^−^_,_ S^2–^)], 100-µL samples were taken from liquid cultures without disturbing the precipitated iron sulfide minerals, and directly transferred to 100 µL of an anoxic 0.2 M NaOH solution. From the alkalinized sample, 10 μL to 20 µL were fixed in 100 µL of a 0.1 M zinc acetate solution, and sulfide was quantified by the methylene blue method (49). The corresponding amount of H_2_S in the headspace was calculated using Henry’s law and a temperature-adjusted k value of 0.093 (28 °C) (50). CH_4_ was measured by gas chromatography with a flame ionization detector (SGI 8610C; SRI Instruments) using a consecutive arrangement of a Porapak (80/100 mesh; 1 m × 2 mm) and a Hayesep-D packed column (80/100 mesh; 3 m × 2 mm). The injector and column temperatures were 60 °C, and the detector temperature was 135 °C. The carrier gas was N_2_ at a flow rate of 3.2 mL⋅min^−1^. Chromatograms were recorded with the PeakSimple v4.44 chromatography software.

Iron-sulfide minerals were analyzed by ^57^Fe Mössbauer spectroscopy. Within an anoxic glove box (100% N_2_), the enrichment culture was passed through a 0.44-µm filter and then sealed between two pieces of air-tight Kapton tape. Samples were transferred to a Mössbauer spectrometer (WissEl) within an airtight bottle filled with 100% N_2_ that was only opened immediately before loading the samples inside the closed-cycle exchange gas cryostat (Janis cryogenics). Measurements were collected at a temperature of 5 K with a constant acceleration drive system (WissEl) in transmission mode with a ^57^Co/Rh source and calibrated against a 7 µm thick α-^57^Fe foil measured at room temperature. All spectra were analyzed using Recoil (University of Ottawa) by applying the Voight Based Fitting (VBF) routine (51). The half width at half maximum (HWHM) was fixed to a value of 0.138 mm s^−1^ for all samples.

XRD patterns were recorded with the D8 Discover system (Bruker) with I µS radiation source (2 mm in diameter), and a Lynxexe XE detector. Samples were dried for 2 h under a continuous stream of 100% N_2_ and measured within 48 h under air as described previously (52). Measurements were done using CuKα rays in angles ranging from 10° to 70° 2Θ in 0.02° steps with 2,880 s measuring time and a total measuring time of 12 h and 47 min. The resulting spectra were compared with spectra provided in the International Crystal Structure Database, FIZ Karlsruhe (version 2016/2) using the software DIFFRAC.EVA (version 4.1.1, Bruker).

### SEM-EDX.

For SEM-EDX analysis, 1 mL of culture was centrifuged at 4,500 × *g* for 10 min. Then 200 µL of the resulting pellet was transferred onto gelatin-coated glass slides. Samples were fixed in 1 mL of 2.5% glutaraldehyde in 0.1 M Hepes buffer containing 0.01 M KCl (Hepes-KCl) and in 2% OsO_4_ in Hepes-KCl for 60 min each. Fixed samples were dehydrated in a graded ethanol series (30%, 50%, 70%, 80%, 90%, 96%, and absolute ethanol) for 30 min each. Thereafter, samples were critical-point dried under CO_2_ in a Bal-Tec CPD030 (Balzers). Sputter coating of 6 nm of platinum was done in a Quorum Q150R ES sputter coater (Quorum Technologies), and micrographs were taken with a FESEM Auriga 40 (Zeiss). EDX mappings and point measurements were taken at a working distance of 5 mm with an Oxford X-Max detector (Oxford Instruments) and at 10 and 15 kV, respectively. Point measurements were normalized to 10,000 counts within a Kα energy of 6.3 keV to 6.5 keV. Sample preparation for cell counts by fluorescent microscopy is described in detail in *SI Appendix*.

### Phylogenetic Analysis.

Total genomic DNA was extracted from 50 mL of a 4.5-mo-old culture using a phenol-based beat-beating protocol modified after Loy et al. (53). Subsequent amplification of bacterial or archaeal 16S rRNA genes was done using standard PCR protocols based on universal primers. Details are given in *SI Appendix*. The 16S rRNA gene clone libraries were constructed using the TOPO TA Cloning Kit (ThermoFisher Scientific). Bacterial or archaeal 16S rRNA gene fragments were aligned by use of the SILVA incremental aligner (SINA) webaligner (54) to the nonredundant 16S rRNA gene database v.123.1 available on the SILVA online platform (55, https://www.arb-silva.de) and imported into the ARB software suite for initial phylogenetic analysis (56). OTU clustering was performed in mothur v.1.22.2 (57) using the furthest-neighbor approach and a 99% identity cutoff to delineate OTUs at the approximate species level (58). For phylogenetic inference of 16S rRNA gene fragments representing individual OTUs, Maximum Likelihood (ML) trees were calculated using RAxML v8.2.9 (59) as implemented on the Cyberinfrastructure for Phylogenetic Research (CIPRES) webserver (60) (www.phylo.org). Using a 50% conservation filter of nucleic acid positions within the domain Bacteria, an RAxML tree was inferred from 1,102 unambiguously aligned nucleic acid positions for bacterial 16S rRNA genes. The reconstruction of the archaeal tree followed the same outline, but using 752 unambiguously aligned nucleic acid positions and no conservation filter because of the close relatedness of all included sequences. Calculations were based on the GTRGAMMA distribution model of substitution rate heterogeneity. Extended majority rule (MRE)-based bootstrap analysis stopped after 204 and 102 replicates for the bacterial and archaeal 16S rRNA gene trees, respectively. Sequences are available from NCBI GenBank under accession numbers MH665848 to MH665880 and MH665881 to MH665889 for Bacteria and Archaea, respectively.

## Supplementary Material

Supplementary File
