# Supplementary material for: Pyrite formation from FeS and H2S is mediated through microbial redox activity
Source: Proc Natl Acad Sci U S A. 2019 Mar 18;116(14):6897–902. doi: 10.1073/pnas.1814412116 (PMC6452648; doi:10.1073/pnas.1814412116)
Supplement: Supplementary File [file pnas.1814412116.sapp.pdf]

Supporting Information for

Pyrite formation from FeS and H_2S is mediated through a novel microbial redox activity

Joana Thiel, James Byrne, Andreas Kappler, Bernhard Schink, Michael Pester

Supporting Materials and Methods

Control experiment for biologically supported abiotic pyrite formation. To test for abiotic pyrite formation mediated unspecifically by cell surfaces or other cell constituents, we incubated anoxic freshwater medium supplemented with 5 mM FeS and 6 mM H_2S with mixtures of alive and dead cells of microorganisms isolated from culture J5. To do so, a *Desulfovibrio* spp, a highly enriched *Desulfomicrobium* spp., and a *Methanospirillum* spp. were isolated from culture J5 using either DSM medium 641, the described freshwater medium supplemented with 10 mM sulfate and 10 mM L-lactate, or the described freshwater medium supplemented with 2 mM Acetate and a H_2/CO_2 (79%/21%) atmosphere, respectively. The three microbial species showed 99.87%, 99.85%, and 100% 16S rRNA gene identity to bacterial OTU 2, bacterial OTU 3, and archaeal OTU1 obtained from culture J5, respectively. Growth was followed by OD_{600} spectroscopic measurements. Cultures of the three species were harvested anoxically in the exponential phase and mixed in the ratio 50:10:40 (*Desulfomicrobium*:*Desulfovibrio*:*Methanospirillum*) as informed by the relative abundances of the respective clones and assuming an 60:40 ratio of bacteria and archaea in culture J5. Cell numbers were calculated with the assumption that OD_{600} of 1 equals $8 \cdot 10^8$ cells mL^{-1} (1). Mixed cultures were centrifuged and re-suspended in anoxic freshwater medium to reach a final densitiy of $1 \cdot 10^8$ cells mL^{-1} and incubated with 5 mM FeS and 6 mM H_2S for 30 days at 28°C. A parallel setup was done with the same mixture of cells but autoclaving it before

www.pnas.org/cgi/doi/10.1073/pnas.1814412116

addition to the freshwater medium supplemented with 5 mM FeS and 6 mM H_2S. Incubations with autoclaved cells were running for 30 days as well. In a third setup, the *Methanospirillum* spp. was mixed with washed cells of *Escherichia coli* strain XL-1 Blue (Agilent, USA) in a ratio of 1:1 yielding $2 \cdot 10^8$ cells mL^{-1}. Incubations were performed as stated above for 28 days. All treatments as well as a parallel incubation of culture J5 were performed in biological triplicates and analyzed using SEM-EDX measurements, Mössbauer spectroscopy, and methane measurements. To counterbalance the effect of shorter incubation times as compared to the normal growth period of culture J5, we used a 10- to 100-fold higher density of alive or dead cells in the supplied mixtures to test for abiotic pyrite formation.

Cell counts by fluorescence microscopy. For cell counts, 0.5 mL culture was fixed overnight in 9.5 mL freshly prepared paraformaldehyde solution (4%), subsequently centrifuged at 10,000 ×*g* for 10 min at 4°C and re-suspended in 1 mL PBS [130 mM NaCl, 5% (v/v) phosphate buffer (40 mM NaH_2PO_4, 160 mM Na_2HPO_4), pH 7.2] and 9 mL ammonium oxalate solution (5.6 g ammonium-oxalate and 4.2 g oxalic acid dihydrate in 200 mL distilled water). Samples were vortexed for 10 min to dissolve most of the iron sulfide minerals so that cells could be collected on a 0.2 µm pore size filter (GTTP-white, Millipore). Filters were air-dried and stored at –20°C. Filter sections were stained with a 1 µg mL^{-1} 4',6-diamidino-2-phenylindole (DAPI) solution and incubated for 10 min in the dark. Thereafter, filters were washed for 5 min in distilled water, followed by two 1-min washing steps in 80% ethanol. Dry DAPI-stained filters were mounted on microscope slides using CitiFluor™ AF1. For fluorescence microscopy, an inverted microscope (AxioObserver, Zeiss) with a 40x/0.60 LD-PlanNeofluar objective was used. Z-stacks were acquired with a distance of 0.28 µm. Image processing involved 3-dimensional deconvolution of each stack using a theoretical PSF with

ZEN Black (Zeiss AG). Cells were counted using an image processing workflow set up in KNIME 3.4.0 (2) using orthogonal projections of the de-convoluted input stacks. The workflow is available at https://github.com/bic-kn/cell-counting-workflow.

DNA extraction. The DNA extraction protocol was adopted from (3). A 4.5-month old 50-mL culture was harvested after CH_4 concentrations reached a plateau of 2.1% in the headspace (corresponding to 55 µmol produced CH_4). Harvesting was done by 10 min of centrifugation at 6,000 ×*g*. The pellet was re-suspended in 400 µL autoclaved TE-Buffer (10 mM Tris & 1 mM EDTA in MQ water, pH 8) and stored for three hours at –20°C. Cells were thawed on ice, mixed with heat-sterilized zirconium beads (0.1 mm), 600 µL phenol/chloroform/isoamylalcohol (25:24:1, Carl Roth), and 150 µL of a 10% sterile-filtered SDS-solution in a screw-cap tube, and vigorously shaken for 20 min using a vortexer. After centrifugation for 20 min at 20,817 ×*g* and 4°C, the aqueous supernatant was transferred to a new reaction tube. Because the aqueous phase was hardly visible due to remaining iron sulfide minerals, another 10-minute centrifugation step was used to remove residual phenol from the extract. DNA was precipitated by incubation at –20°C overnight in 0.1 volume of 3 M Na-acetate (in MQ-water, autoclaved) and 2.5 volumes absolute ethanol. Afterwards, the pellet was washed twice with 70% ethanol, dried for 5 min, and re-suspended in 50 µL DNase- and RNAse- free H_2O. DNA concentrations were quantified fluorimetrically using Quant-iT PicoGreen (Invitrogen).

16S rRNA gene clone library. Amplification of bacterial 16S rRNA genes was performed with Bact8f (5'-AGA GTT TGA TYM TGG CTC-3') as forward primer (4) and 1492r (5'-N TAC CTT GTT ACG ACT-3') as reverse primer (5). Archaeal species were targeted by AR109F (5'-ACK GCT CAG TAA CAC GT-3') as forward (6) and AR915 (3'-GTG CTC CCC CGC CAA TTC CT-3') as reverse primer (7). The PCR mixture contained 0.2 mM of each dNTP, 2 mM $MgCL_2$, 20

µg BSA, 1 U of *Taq* DNA polymerase, and a Taq polymerase buffer with KCl (ThermoFisher Scientific). The PCR was performed using an initial denaturation at 95°C for 5 min; 30 cycles of 95°C for 30 s, 50°C for 30 s, 72°C for 1.5 min; and a final elongation at 72°C for 7 min. For PCRs with archaeal primers, the annealing temperature was set to 55°C. Amplification products were purified by use of the Zymo Research DNA Clean & Concentrator Kit (Zymo Research). 16S rRNA clone libraries were obtained with the TOPO® TA Cloning® Kit (ThermoFisher Scientific). Clones were screened by M13-PCR for inserts of the correct size according to the manufacturer's instructions. Resulting PCR products of expected length were purified by use of the Zymo Research DNA Clean & Concentrator Kit and sent for sequencing.

Supporting Tables

Table S1. Overview of inocula used to establish initial pyrite-forming enrichment cultures.

Enrichment	Date	Sampling area	Material	Medium†	Temp. (°C)	pH
J5*	Sept. 1995	Sewage treatment plant Konstanz, Germany	digested sewage sludge	freshwater	28	7.2
J2*	Sept. 1995	Sewage treatment plant Konstanz, Germany	digested sewage sludge	freshwater	16	7.2
J7	Apr. 1993	Rio Tentor (Venice, Italy)	brackish sediment	marine	16	7.2
J8*	Mar. 1991	Wadden sea sediment, Groningen, The Netherlands	marine sediment	marine	16	7.2
J9*	Apr. 1993	Fish market channel (Venice, Italy)	brackish sediment	marine	16	7.2
X1	Sept. 1995	Sewage treatment plant Tübingen-Lustnau, Germany	digested sewage sludge	freshwater	28	7.2
X2	Sept. 1995	Lake Constance, Güll	freshwater sediment	freshwater	28	7.2

† after Widdel and Pfennig (8)
* CH_4 formation observed for more than ten transfers

Table S2. Iron mineral analysis by Mössbauer spectroscopy at a temperature of 5 K in nearly 7-month-old (207 days) culture J5 incubated at various temperatures and in the presence of various inhibitors. Mössbauer parameters were obtained through Voigt based fitting (VBF). δ – isomer shift, ΔE_Q – quadrupole splitting, ε – quadrupole shift, B_{hf} – internal magnetic field, R.A. – relative area, X^2 – goodness of fit parameter. The absolute amount of formed FeS_2 was inferred from the relative area of the FeS_2 signal and the maximum amount of 350 µmol that could be produced if all FeS would have been converted to FeS_2.

**Sample**	**Phase**	**δ (mm/s)**	**ΔE_Q (mm/s)**	**ε (mm/s)**	**B_{hf} (T)**	**R. A. %**	**X^2**	**FeS_2 (µmol)**
Abiotic	FeS_x	0.43		0.15	13.1	64.3	1.67	
control	FeS	0.50	0.13			35.7		
4 °C	FeS	0.47	0.27			37.8	6.01	
	FeS_x	0.43		0.07	17.2	62.2		
16 °C	"FeS_2-FeS"‡	0.41	0.38			18.5	0.78	64.8
	FeS_x	0.39		0.02	19.2	48.8		
	FeS_x	0.42		–0.02	16.5	32.7		
28 °C	FeS_2	0.41	0.50			52.8	0.88	184.8†
Replicate I	FeS_x	0.32		0.07	15.7	38.9		
	Fe_3S_4	0.46		0.05	32.0	8.3		
28 °C	FeS_2	0.40	0.58			62.5	0.78	218.8§
Replicate II	FeS_x	0.29		–0.2	16.4	31.0		
	Fe_3S_4	0.60		–0.05	32.0	6.5		
46 °C	"FeS_2-FeS" ‡	0.42	0.41			39.4	1.67	137.9
	FeS_x	0.10		–0.09	21.1	50.5		
	FeS_x	0.40		0.05	15.7	7.8		
	Fe_3S_4	0.64		0.03	33.2	2.3		
60 °C	FeS	0.54	0.16			27.1	1.74	
	FeS_x	0.34		0.05	2.4	21.6		
	FeS_x	0.55		–0.08	16.6	43.9		
	Fe_3S_4	0.63		–0.08	31.9	7.3		
Penicillin + 79% H_2	FeS	0.51	0.16			31.6	1.12	
(headspace)	FeS_x	0.38		0.15	5.3	17.4		
	FeS_x	0.85		0.01	23.0	37.5		
	FeS_x	0.46		–0.07	15.5	13.4		
Penicillin	FeS	0.46	0.27			26.4	1.15	
	Fe_3S_4	0.68		–0.04	33.1	8.8		
	Fe_3S_4	0.52		0.05	31.7	18.8		
	FeS_x	0.44		0.00	14.9	15.0		
	FeS_x	0.43		0.02	20.2	31.1		
BES	FeS	0.50	0.22			28.1	0.76	
	Fe_3S_4	0.69		–0.15	29.6	23.4		
	Fe_3S_4	0.62		0.02	33.1	7.1		
	FeS_x	0.25		0.15	7.4	20.2		
	FeS_x	0.44		–0.16	16.6	21.2		

† corresponding amount of formed CH_4: 44.9 µmol
§ corresponding amount of formed CH_4: 67.6 µmol
‡ an yet undefined intermediate FeS_2-FeS phase

Supporting Figures

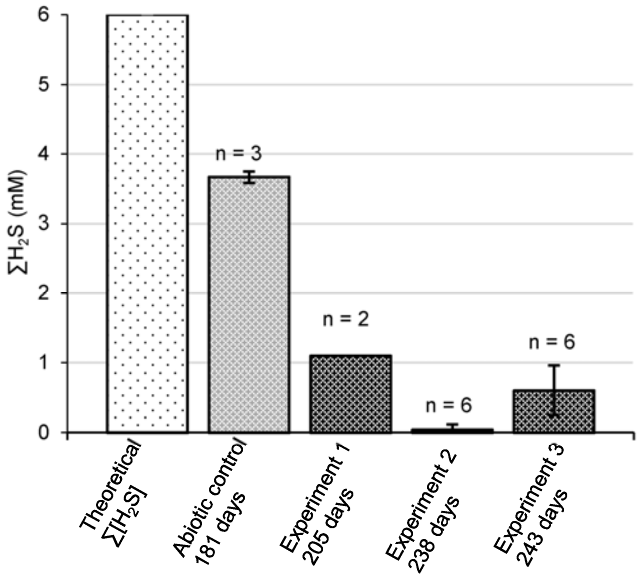

Figure S1. Total H_2S as the sum of $H_2S_{gaseous}$, $H_2S_{aqueous}$, HS^{-}, and S^{2-} in the non-inoculated medium as compared to the abiotic control and enrichment culture J5 in various independent incubation experiments. The time of incubation is indicated in days. Biological replicates are indicated as n.

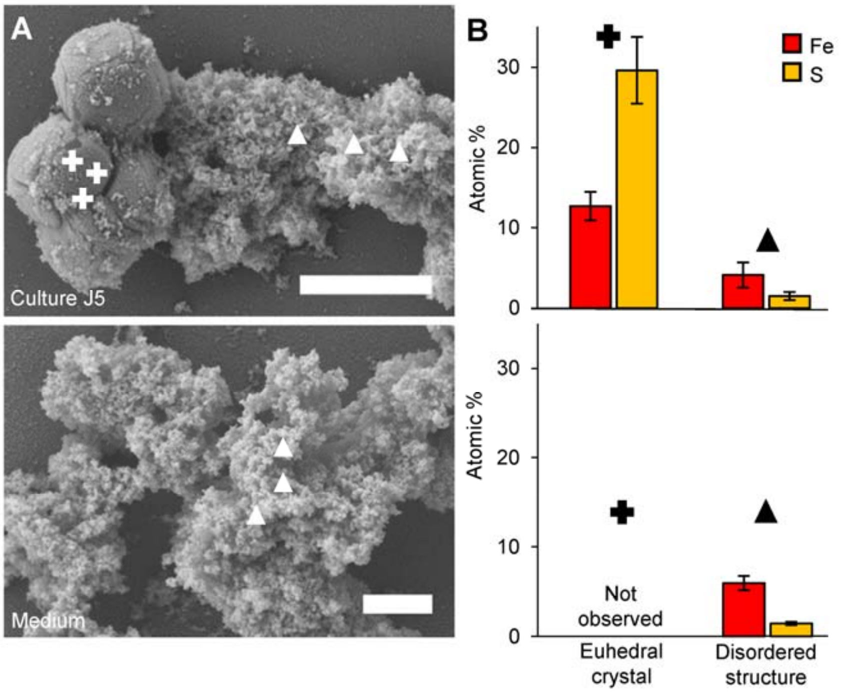

Figure S2. Fe:S ratio of different mineral phases in culture J5 and freshly prepared medium without inoculum. (A) Exemplary scanning electron microscopy images used as guidance to perform energy dispersive X-ray spectroscopy (EDX) point measurements of culture J5 after nearly 10 months of incubation (295 days) and of freshly prepared medium without inoculum. Scale bars represent 2 µm. Symbols in the SEM images indicate EDX point measurements (crosses for crystals, triangles for disordered structure). (B) Atom percent ratio of iron (red) and sulfur (yellow) as derived from EDX point measurements of euhedral crystals resembling pyrite as well as disordered structures resembling the sum of the remaining Fe-S-mineral phase. Measurements were done on eight different sampling areas with three EDX point measurements each.

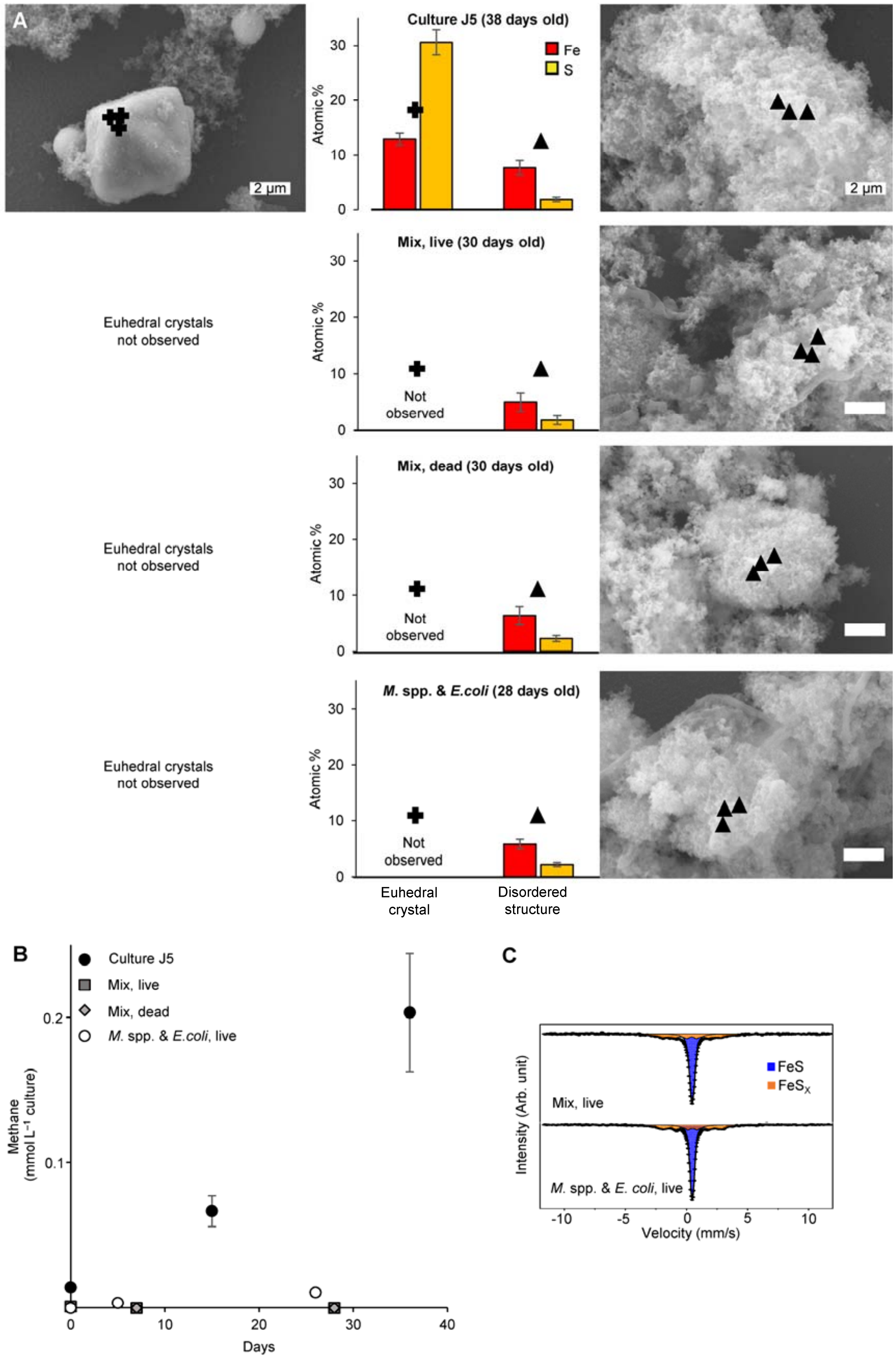
A
Culture J5 (38 days old)
Fe
S
Atomic %
2 µm
Mix, live (30 days old)
Euhedral crystals not observed
Not observed
Mix, dead (30 days old)
Euhedral crystals not observed
Not observed
M. spp. & E.coli (28 days old)
Euhedral crystals not observed
Not observed
Euhedral crystal
Disordered structure
B
Culture J5
Mix, live
Mix, dead
M. spp. & E.coli, live
Methane (mmol L⁻¹ culture)
Days
C
FeS
FeSx
Mix, live
M. spp. & E. coli, live
Intensity (Arb. unit)
Velocity (mm/s)

Fig. S3. Pyrite and methane formation in culture J5 in comparison to incubations inoculated with living (mix, live) or autoclaved (mix, dead) mixtures of a *Desulfomicrobium* spp., *Desulfovibrio* spp., and *Methanospirillum* spp. or a mixture of living *Escherichia coli* and a Methanospirillum spp. (*M. spp* & *E.coli*). (A) Exemplary scanning electron microscopy images taken at the indicated time point and used as guidance to perform energy dispersive X-ray spectroscopy (EDX) point measurements for seven images per culture. Symbols in the SEM images indicate EDX point measurements (crosses for crystals, triangles for disordered structure). Bar charts in the middle show the atom percent ratio of iron (red) and sulfur (yellow) as derived from EDX point measurements of euhedral crystals resembling pyrite as well as disordered structures resembling the sum of the remaining Fe-S-mineral phase. (B) Time-resolved CH_4 formation over the whole incubation period. (C) Mössbauer spectra corresponding to the SEM-EDX measurements of the incubations "mix, live" and "*M. spp.* & *E.coli*, live".

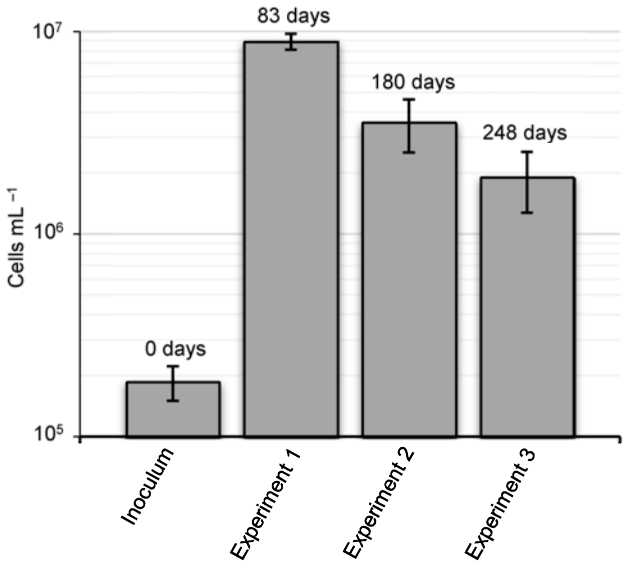

Figure S4. Average cell counts of culture J5 as based on DAPI-stained cells in three independent incubation experiments at 28°C as compared to freshly inoculated medium. The time of incubation is indicated in days. Data was obtained from biological duplicates, each measured in technical triplicates. Standard deviations are given for technical replicates.

References

1. Sezonov G, Joseleau-Petit D, D'Ari R (2007) *Escherichia coli* physiology in Luria-Bertani Broth. *J. Bacteriol.* 189(23):8746-8749.

2. Berthold MR*, et al.* (2008) KNIME: The Konstanz Information Miner. (Springer), pp 319-326.

3. Loy A, Beisker W, Meier H (2005) Diversity of bacteria growing in natural mineral water after bottling. *Appl Environ Microbiol* 71(7):3624-3632.

4. Loy A*, et al.* (2002) Oligonucleotide microarray for 16S rRNA gene-based detection of all recognized lineages of sulfate-reducing prokaryotes in the environment. *Appl Environ Microbiol* 68(10):5064-5081.

5. Berry D, Ben Mahfoudh K, Wagner M, Loy A (2011) Barcoded primers used in multiplex amplicon pyrosequencing bias amplification. *Appl Environ Microbiol* 77(21):7846-7849.

6. Lueders T, Friedrich MW (2002) Effects of amendment with ferrihydrite and gypsum on the structure and activity of methanogenic populations in rice field soil. *Appl Environ Microbiol* 68(5):2484-2494.

7. Casamayor EO*, et al.* (2002) Changes in archaeal, bacterial and eukaryal assemblages along a salinity gradient by comparison of genetic fingerprinting methods in a multipond solar saltern. *Environ Microbiol* 4(6):338-348.

8. Widdel F, Pfennig N (1981) Studies on dissimilatory sulfate-reducing bacteria that decompose fatty acids. I. Isolation of new sulfate-reducing bacteria enriched with acetate from saline environments. Description of *Desulfobacter postgatei* gen. nov., sp. nov. *Arch Microbiol* 129(5):395-400.
